# The health needs of people leaving prison with a history of methamphetamine and/or opioid use

**DOI:** 10.1111/dar.13636

**Published:** 2023-03-14

**Authors:** Craig Cumming, Stuart A. Kinner, Rebecca McKetin, Ian Li, David B. Preen

**Affiliations:** ^1^ Centre for Health Services Research, School of Population and Global Health University of Western Australia Perth Australia; ^2^ Centre for Adolescent Health Murdoch Children's Research Institute Melbourne Australia; ^3^ Melbourne School of Population and Global Health The University of Melbourne Melbourne Australia; ^4^ Griffith Criminology Institute Griffith University Brisbane Australia; ^5^ School of Population Health Curtin University Perth Australia; ^6^ National Drug and Alcohol Research Centre UNSW Sydney Sydney Australia; ^7^ School of Population and Global Health University of Western Australia Perth Australia

**Keywords:** harm reduction, methamphetamine, opioid, prisons, substance‐related disorders

## Abstract

**Introduction:**

Methamphetamine use is more common than opioid use among prison entrants in some countries, including Australia, yet most research and policy focuses on opioid use. This suggests that traditional opioid‐focused interventions are no longer appropriate for the majority of this group in countries such as Australia. To inform policy and practice, we compared socio‐demographic characteristics and health needs of people leaving prison with a history of methamphetamine use and/or opioid use.

**Methods:**

A cross‐sectional survey of incarcerated adults administered the World Health Organization Alcohol, Smoking and Substance Involvement Screening Test was used to identify moderate‐/high‐risk methamphetamine use (*n* = 909), opioid use (*n* = 115) or combined methamphetamine/opioid use (*n* = 356) before incarceration. We compared groups using modified log‐linked Poisson regression with robust error variance.

**Results:**

Compared to the opioid‐only group, the methamphetamine‐only group were: significantly more often aged <25 years; significantly more likely to identify as Indigenous; significantly less likely to have a history of prior incarceration, drug injection or overdose. A significantly lower proportion of methamphetamine‐only and methamphetamine‐and‐opioid participants self‐reported current hepatitis C infection compared to opioid‐only participants. A majority of participants in all groups screened positive for current psychological distress according to the K10.

**Discussion and Conclusions:**

People leaving prison with a history of methamphetamine use differ from opioid users with respect to demographics, patterns of substance use and related health concerns. Treatment and harm reduction efforts for people who experience incarceration must respond to patterns of drug use in this population, and invest at scale in coordinated, continuous services for co‐occurring substance use and mental health problems.

**Key Point Summary**

- Methamphetamine use is many times more prevalent than opioid use among people entering prison in Australia and other countries.
- A history of injecting drug use is reported by people in prison who have used methamphetamine and/or opioids in the past.
- Using opioid substitution therapies to reduce the risk of blood‐borne virus transmission among people in prison will likely miss a large proportion of injecting drug users.
- Increased treatment and support for both substance use and mental illness that commence in prison and continue after release into the community are urgently needed.

## 1 INTRODUCTION

There is growing evidence of the adverse health impacts of methamphetamine use [1, 2], including increased numbers of methamphetamine‐related deaths [3]. The use of methamphetamine has also become more prominent among people who go to prison in some countries [4] and rates of methamphetamine use are often many times higher than opioids among this group [5, 6]. Despite this, prison health literature overwhelmingly focusses on opioid use, including related deaths [7], injecting drug use [8], the transmission of blood‐borne viruses (BBV) [9] and opioid substitution therapy [10].

More information is needed on the use of methamphetamine among people who experience incarceration to help to inform treatment strategies, relapse prevention and harm reduction efforts. To address this knowledge gap we analysed data from the Health After Release from Prison study: the largest ever prospective cohort of adults transitioning from prison to the community in Australia [11]. We compared the socio‐demographic characteristics and health status (BBV exposure, overdose, mental health) for people who had used opioids, methamphetamine or both drugs at moderate to high‐risk levels in the 3 months prior to incarceration.

## 2 METHODS

### 2.1 Procedure and participants

This is a cross‐sectional study using data from the baseline survey of a cohort of adults prior to their release from prison. Participants were recruited from seven prisons in Queensland between 2008 and 2010, and five prisons in Western Australia between 2013 and 2016. Adults (18 years and over, including both males and females) who were incarcerated for at least 4 weeks, and expected to be released within 6 weeks, were invited to participate in the study. Of the 3867 individuals invited, 2698 consented, a recruitment fraction of 69.8%. Those consenting then completed the baseline survey at some point in the 6 weeks prior to their expected release from prison. All individuals provided informed consent prior to participation. The survey was administered face‐to‐face by trained research staff not affiliated with corrective services and took 60–90 min to complete. Participants were not offered any incentive to participate in the baseline survey.

During the baseline survey, we assessed substance use in the 3 months prior to incarceration using the World Health Organization Alcohol, Smoking and Substance Involvement Screening Test (ASSIST) [12]. Participants were requested to think back to their drug use in 3 months before they were incarcerated when completing the ASSIST items. Based on their responses to the ASSIST, only participants identified as having engaged in moderate or high‐risk methamphetamine and/or opioid use (defined as a score of ≥4 on the ASSIST) in the 3 months before they were incarcerated were included in this analysis (*n* = 1380).

### 2.2 Additional measures

#### 2.2.1 Socio‐demographic characteristics

At baseline age, Indigenous status, previous incarceration, current relationship status and employment/housing status in the 6 months before incarceration were self‐reported. Sex was inferred from the prison where participants were recruited.

#### 2.2.2 Mental health

Participants self‐reported lifetime diagnoses of mental disorder (diagnosed by a doctor, psychologist or psychiatrist) in response to questions adapted from the Australian National Health Survey [13]. Those responding in the affirmative indicated if they had been diagnosed with anxiety, depression, any substance use disorder or schizophrenia.

Current psychological distress at baseline was assessed using the Kessler Psychological Distress Scale [14]. Participants were classified as experiencing low/no psychological distress (score of <16) or moderate/high/very high distress (score of ≥16), consistent with the National Health Survey [15].

#### 2.2.3 BBV and sexually transmitted infections

Participants were asked if they were currently infected with a BBV: hepatitis A, hepatitis B, hepatitis C virus (HCV) and/or HIV. Participants were also asked if they ever had any of the following sexually transmitted infections (STI): gonorrhoea, genital warts, genital herpes, chlamydia or syphilis (combined to create a single STI variable).

#### 2.2.4 BBV risk behaviours and overdose

Participants were asked if they had ever injected drugs, injected drugs in prison, injected during their current period of incarceration, shared injecting equipment, shared injecting equipment in prison and/or received a high‐risk tattoo (been tattooed outside of a licensed or regulated tattooing premises). Participants were also asked whether they had ever self‐harmed and whether they had ever overdosed or become unconscious as a result of taking drugs.

### 2.3 Statistical analysis

Participants were assigned to one of three mutually exclusive groups: (i) methamphetamine use only (“meth‐only” group); (ii) opioid use only (“opioid‐only” group, the reference group); and (iii) methamphetamine and opioid use (“meth‐and‐opioid” group). All other variables were either binary or re‐coded as binary for analysis. We performed univariate modified log‐linked Poisson regression with robust error variance to compute the relative risk (RR) of the opioid‐only group to each other group, as previously recommended [16]. We used Stata version 16 [17] for all statistical analyses.

## 3 RESULTS

Of the 1380 eligible participants, 23% (*n* = 315) were female and 77% (*n* = 1065) were male. We assigned 909 (66%) to the meth‐only group, 115 (8%) to the opioid‐only group and 356 (26%) to the meth‐and‐opioid group based on their responses to the ASSIST, relating to their use of each drug in the 3 months prior to incarceration. The majority of the meth‐only group had been incarcerated prior to their current period of incarceration (74%), had ever injected drugs (79%) and had ever engaged in high‐risk tattooing (59%), with a minority having been employed in the 6 months before prison (33%) or in a current stable relationship at baseline (36%) (Table 1).

**TABLE 1:** Socio‐demographic characteristics, BBV/overdose risk behaviours, mental health, BBV and STI.

	Opioid‐only (%), *n* = 115	Meth‐only (%), *n* = 909	RR (95% CI)	*p*‐value	Meth. and opioid (%), *n* = 356	RR (95% CI)	*p*‐value	Total (%), *N* = 1380
Socio‐demographic								
<25 years old	15 (13)	317 (35)	2.67 (1.65, 4.32)	<0.001	75 (21)	1.62 (0.97, 2.70)	0.067	407 (29)
Female (%)	30 (26)	192 (21)	0.81 (0.58, 1.13)	0.213	93 (26)	1.00 (0.70, 1.43)	0.994	315 (23)
Indigenous (%)	22 (19)	350 (39)	2.01 (1.37, 2.96)	<0.001	93 (26)	1.37 (0.90, 2.07)	0.141	465 (34)
Previously incarcerated (%)	96 (84)	665 (74)	0.87 (0.80, 0.95)	0.003	311 (88)	1.04 (0.95, 1.14)	0.382	1072 (78)
Stable current relationship (%)	50 (43)	331 (36)	0.84 (0.67, 1.05)	0.123	129 (36)	0.83 (0.65, 1.07)	0.153	510 (37)
Employed before prison (%)	39 (34)	304 (33)	0.99 (0.75, 1.29)	0.920	100 (28)	0.82 (0.61, 1.12)	0.225	443 (32)
Stable housing before prison (%)	101 (88)	808 (89)	1.01 (0.94, 1.09)	0.743	306 (86)	0.98 (0.90, 1.06)	0.598	1215 (88)
BBV/overdose risk behaviours								
Ever injected drugs	110 (96)	719 (79)	0.83 (0.79, 0.87)	<0.001	344 (97)	1.01 (0.97, 1.06)	0.647	1173 (85)
Ever shared needle/syringe	82 (71)	328 (36)	0.51 (0.44, 0.59)	<0.001	275 (77)	1.08 (0.95, 1.23)	0.224	685 (50)
Ever injected in prison	57 (50)	204 (22)	0.45 (0.36, 0.56)	<0.001	214 (60)	1.21 (0.99, 1.49)	0.062	475 (34)
Injected during current incarceration	36 (31)	131 (14)	0.46 (0.34, 0.63)	<0.001	141 (40)	1.27 (0.94, 1.71))	0.124	308 (22)
Ever shared needle/syringe in prison	34 (30)	125 (14)	0.47 (0.34, 0.64)	<0.001	132 (38)	1.29 (0.94, 1.76)	0.113	291 (21)
Ever overdosed (lost consciousness)	68 (59)	222 (24)	0.41 (0.34, 0.50)	<0.001	203 (57)	0.96 (0.81, 1.15)	0.687	493 (36)
Risky tattooing	68 (59)	539 (59)	1.00 (0.85, 1.18)	0.973	234 (66)	1.11 (0.94, 1.32)	0.221	841 (61)
Mental health								
Any mental illness (including SUD)	65 (57)	437 (48)	0.85 (0.71, 1.01)	0.068	229 (65)	1.14 (0.96, 1.36)	0.146	731 (53)
Anxiety	27 (23)	131 (14)	0.61 (0.43, 0.89)	0.009	99 (28)	1.18 (0.82, 1.71)	0.370	257 (19)
Depression	33 (29)	245 (27)	0.94 (0.69, 1.28)	0.690	136 (38)	1.33 (0.97, 1.83)	0.077	414 (30)
SUD	26 (23)	73 (8)	0.36 (0.24, 0.53)	<0.001	71 (20)	0.88 (0.59, 1.31)	0.536	170 (12)
Schizophrenia	7 (6)	66 (7)	1.19 (0.56, 2.54)	0.647	43 (12)	1.98 (0.92, 4.29)	0.081	116 (8)
Moderate/high distress (K10)	66 (57)	537 (59)	1.03 (0.87, 1.22)	0.734	209 (59)	1.03 (0.86, 1.23)	0.781	812 (59)
Ever self‐harmed	19 (17)	164 (18)	1.09 (0.71, 1.69)	0.687	67 (19)	1.14 (0.72, 1.81)	0.582	250 (18)
Ever attempted suicide	25 (22)	247 (27)	1.25 (0.87, 1.80)	0.228	102 (29)	1.32 (0.90, 1.93)	0.158	374 (27)
BBV and STI								
Any STI	19 (17)	226 (26)	1.50 (0.98, 2.29)	0.063	83 (24)	1.40 (0.89, 2.19)	0.145	328 (24)
Hepatitis C	67 (58)	162 (18)	0.31 (0.25, 0.38)	<0.001	171 (48)	0.82 (0.68, 1.00)	0.045	400 (29)
Among injectors only (*n* = 1173)	67 (61)	160 (22)	0.37 (0.30, 0.45)	<0.001	171 (50)	0.82 (0.68, 0.98)	0.030	398 (34)

Abbreviations: BBV, blood‐borne virus; CI, confidence interval; K10, Kessler Psychological Distress Scale; Meth., methamphetamine; RR, relative risk; STI, sexually transmitted infection—includes gonorrhoea, genital warts, herpes, chlamydia, syphilis; SUD, substance use disorder.

The meth‐only group were significantly more likely than the opioid‐only group to be under 25 years old at baseline (RR = 2.67; 95% confidence interval [CI] 1.65, 4.32), Indigenous (RR = 2.01; 95% CI 1.37, 2.96) and significantly less likely to have been previously incarcerated (RR = 0.87; 95% CI 0.80, 0.95).

The meth‐only group were significantly less likely than the opioid‐only group to have ever: injected drugs (RR = 0.83; 95% CI 0.79, 0.87), shared a needle/syringe (RR = 0.51; 95% CI 0.44, 0.59), injected in prison (RR = 0.45; 95% CI 0.36, 0.56), injected during their current period of incarceration (RR = 0.46; 95% CI 0.34, 0.63) or shared a needle/syringe in prison (RR = 0.47; 95% CI 0.34, 0.64). The meth‐only group were also significantly less likely than the opioid‐only group to have ever overdosed on drugs (RR = 0.41; 95% CI 0.34, 0.50). Both the meth‐only (RR = 0.31; 95% CI 0.25, 0.38) and meth‐and‐opioid (RR = 0.82; 95% CI 0.68, 1.00) groups were significantly less likely than the opioid‐only group to report a current HCV infection. Just under a quarter (24%) of participants reported ever having an STI, with a (non‐significantly) higher proportion of both the meth‐only (26%) and meth‐and‐opioid (24%) groups reporting ever having an STI compared to the opioid‐only group (17%).

A majority (53%) of participants reported ever receiving a mental illness diagnosis, with the meth‐and‐opioid group having the highest proportion (65%), and the meth‐only group the lowest (48%). There was no significant difference between either methamphetamine group and the opioid‐only group. A majority (59%) of meth‐only participants screened for moderate/high distress on the Kessler Psychological Distress Scale at baseline; this was not significantly different from the opioid‐only group.

## 4 DISCUSSION

In our cohort, a history of moderate‐/high‐risk methamphetamine use was far more common than a history of moderate‐/high‐risk opioid use. A majority reported diagnosis of a mental disorder and most reported significant psychological distress at baseline. Participants with a history of methamphetamine use were younger, more likely to be Indigenous, and less likely to inject drugs or have overdosed than their opioid‐using counterparts. Nevertheless, most of these individuals require coordinated, continuous, multidisciplinary care to address underlying physical health, mental health, substance use and psychosocial needs.

Echoing previous Australian research [6], we confirmed that methamphetamine is the primary substance of concern among the majority of people entering Australian prisons, with a high proportion reporting a history of both methamphetamine use and mental illness, as well as poor psychosocial profiles. We observed substantially fewer participants reporting injecting drug use during their current sentence than those who reported ever injecting drugs, suggesting that drug use likely reduces for many during prison. The implications of this are that tolerances may decrease so that the risk of overdose is high if drug use resumes after prison, as has been observed previously [18, 19, 20]. This, along with the high rates of mental illness we observed, means a tailored multi‐disciplinary response is needed in the period after release from prison to address likely dual diagnosis, as well as providing welfare and psychosocial support at scale in the period after release from prison; aimed at preventing the poor post‐release drug‐related health outcomes that often occur. Improving the co‐ordination of mental health and alcohol and other drug services is essential, as the siloing of these services is a perennial issue [21].

Current HCV infection prevalence was high across our sample, and while meth‐only participants were the group with the lowest prevalence, this was still more than 18 times higher than Australian general population estimates [22]. However as the meth‐only cohort were significantly younger than their opioid‐using counterparts, and the vast majority reported injecting drug use, it could be a ‘matter of time’ before they become infected. The high prevalence of current HCV infection across our sample highlights the importance of BBV prevention in this population; however, our findings suggesting that current opioid substitution therapy interventions [10] likely miss the large number of people using methamphetamine requiring assistance to reduce injecting, so alternatives need to be considered.

Although we did not find a significant difference between either of our methamphetamine groups and opioid‐only group with respect to reporting a lifetime STI, the proportion of participants in the methamphetamine groups were at least 1.5 times higher than estimates from the Australian population [23]. Given evidence that people who inject methamphetamine are at an increased risk of contracting an STI [24], additional support to address both injecting methamphetamine use and high‐risk sexual behaviour is likely warranted for this group.

Treating people using opioids and methamphetamine concurrently is an ongoing challenge, with mental health morbidity highest among this group in our study. Increases internationally in concurrent opioid and methamphetamine use [25] and fatal and non‐fatal overdoses involving these two drugs [26] highlight the ongoing challenges faced in preventing harm for this group. One area of urgent need is for effective pharmacotherapy options for this group, with a dearth of evidence in the area, consisting of a sole randomised controlled trial investigating the use of naltrexone to treat people with co‐occurring methamphetamine and opioid use disorder showing modest efficacy [27, 28]. Furthermore, the poor efficacy of opioid substitution therapy when opioid use is accompanied by methamphetamine use [29], highlights the urgent need for further research to inform improved treatment options, including pharmacotherapy, for people using these drugs together. This is crucial given that the meth‐and‐opioid group outnumbered the opioid‐only group by 3:1 in our study.

When planning services, the younger age and larger proportion of Indigenous participants in our meth‐only group compared to the opioid use groups should be considered. Given the higher proportion of Indigenous participants with a history of moderate‐/high‐risk methamphetamine than opioid use in our study, a failure to respond to demand for methamphetamine use treatment risks compounding health and social inequities experienced by Indigenous people, who are over‐represented in the criminal justice system [30, 31].

Strengths of our study include the large, multi‐jurisdictional sample and our use of the validated ASSIST to assess moderate‐/high‐risk drug use. A limitation was reliance on self‐report data. Recall bias and social desirability bias, particularly where stigmatised behaviours such as drug use are involved, may result in under‐reporting; however, a previous review has found that self‐reports of drug use and drug‐related problems can be reliable and valid [32]. Self‐report data on HCV infection should be interpreted with caution because these may be influenced by testing rates, which may be lower among people who use methamphetamine when compared to opioids, and our findings are inconsistent with other research [33]. For non‐custodial settings, only lifetime injecting drug use data were available, so it was not possible to ascertain how close to the commencement of incarceration participants were injecting. Finally, we only recruited participants from two Australian states to our results may not be generalisable across Australia; however, these two states account for around 39% of all prisoners (and 47% of all Indigenous prisoners) in Australia [30].

## 5 CONCLUSION

A history of moderate‐/high‐risk methamphetamine use is far more common than opioid use among people in Australian prisons. A majority of people who use methamphetamine and/or opioids have a history of mental illness, with those using methamphetamine and opioids concurrently having the most complex mental health and BBV profile. Multi‐disciplinary support is urgently needed to respond to concurrent alcohol and other drug use and mental illness in this group both during incarceration, and as they transition back into the community.

## AUTHOR CONTRIBUTIONS

Each author certifies that their contribution to this work meets the standards of the International Committee of Medical Journal Editors. Craig Cumming led collection of the data for the West Australian arm of the study as well as the conception of the study design, data analysis, interpretation of the data, and drafting of the manuscript. David Preen (Western Australia) and Stuart Kinner (Queensland) oversaw data collection for the study and along with Rebecca McKetin provided feedback on the study design, interpretation of the data, and contributed to the editing of the manuscript. Ian Li provided feedback on the draft manuscript and interpretation of the data and contributed to the editing of the manuscript.

## CONFLICT OF INTEREST STATEMENT

The authors report no conflicts of interest. This research was funded by two grants from the Australian National Health and Medical Research Council (Grant #409966 and Grant #1002463).

## ETHICS STATEMENT

Ethics approval for this study was obtained from The University of Queensland's Behavioural and Social Sciences Ethical Review Committee (Project 2007000607), the Queensland Health Human Research Ethics Committee, The University of Western Australia's Human Research Ethics Committee (Project 2019/RA/4/20/6492 replacing RA/4/1/5076) and the West Australian Aboriginal Health Ethics Committee (Reference number: 370). Research approval was obtained from the Queensland Corrective Services Research Committee and the West Australian Department of Justice's Research Applications and and Advisory Committee.
